# ﻿*Oreochariscorallodiscoides* (Gesneriaceae, Trichosporeae, Didymocarpinae), a new species from Yunnan, southwest China

**DOI:** 10.3897/phytokeys.256.148644

**Published:** 2025-05-13

**Authors:** Xi Li, Qiu-Ping Wang, Feng Yang, Jing-Yi Ye, Huan-Chong Wang

**Affiliations:** 1 School of Ecology and Environmental Science, Yunnan University, Kunming 650091, China Yunnan University Kunming China; 2 Herbarium of Yunnan University, Kunming 650091, Yunnan, China Herbarium of Yunnan University Kunming China

**Keywords:** Dry-hot valley, endemism, morphological comparison, taxonomy, xerophyte

## Abstract

*Oreochariscorallodiscoides*, a new species of the tribe Trichosporeae (Gesneriaceae), is described and illustrated. This new species is characterized by its ovate, rounded or sometimes rhombic leaves densely covered with appressed hairs, yellow corolla, and the presence of two purple triangular appendages inside the base of the corolla tube. It is known only from the type locality, Luzhijiang dry-hot valley in Yimen county, central Yunnan, southwest China. A morphological comparison with its morphologically similar species (*O.agnesiae* and *O.longifolia*) is also presented.

## ﻿Introduction

The genus *Oreocharis* Benth. was first established by the British botanist G. Bentham (1876). For a long time, it was regarded as a medium-sized genus comprising approximately twenty to thirty species (Wang et al. 1998). Based on molecular data and morphological evaluation, Möller et al. (2011) expanded the delimitation of *Oreocharis* by incorporating ten small-sized or monotypic genera (e.g. *Ancylostemon* Craib, *Dayaoshania* W.T.Wang, *Isometrum* Craib, *Paraisometrum* W.T. Wang, *Thamnocharis* W.T.Wang, etc.) into a newly delimited *Oreocharis.* Additionally, several species previously placed in *Briggsia* Craib were transferred to *Oreocharis*, thereby expanding the genus to comprise approximately 90 species (Chen et al. 2014; Möller et al. 2014). The revised genus is characterized by rosette-forming herbs with spirally arranged leaves, scapose inflorescences, and loculicidally dehiscent capsules (Möller et al. 2011). Beyond these shared traits, members of *Oreocharis* exhibit considerable variation in flower morphology (Möller et al. 2011). The treatment of Möller et al. (2011) is generally accepted by recent researchers, such as Fu et al. (2019), Tan et al. (2020), Yang et al. (2020), Li et al. (2023), Xie et al. (2024) and others. After the generic redefinition, about 70 species (Möller 2019; Cai et al. 2020; Chen et al. 2020; Yang et al. 2020) of *Oreocharis* have been described and officially published. Currently, the genus *Oreocharis* contains approximately 160 species (GRC 2025). This genus is mainly distributed in southern and southwestern China, with a few species extending into or occurring in northern Vietnam, India, Bhutan, Japan, Thailand and Myanmar (Möller et al. 2011; Cai et al. 2020). Since 2011, approximately 70 new species of *Oreocharis* have been continuously discovered and published (e.g. Cai et al. 2019, 2020; Chen et al. 2020; Yang and Shi 2021; and summarized in Möller 2019; Wen et al. 2025). Most of these new species are from China, highlighting the need for continued investigation and study of *Oreocharis* diversity in China.

During botanical fieldwork in the Luzhijiang valley, Yimen county, Yunnan province, southwest China, in September 2015, we discovered and collected an unknown species of Gesneriaceae. After a detailed comparison with morphologically similar species, we identified it as a member of *Oreocharis* based on its inflated corolla tube, widely open corolla, and yellow floral coloration—features shared with *O.longifolia* (the type species of the genus *Briggsia*, now placed in *Oreocharis*), and this plant represents a distinct new species.

## ﻿Materials and methods

The study followed standard practices in plant taxonomic surveys and herbarium research. Morphological analyses of the new species were examined through observations of living plants and herbarium specimens. Observation of live plants was carried out on the field population, with approximately 30 individuals directly analyzed and studied. Meanwhile, fresh flowers were brought to the laboratory for dissection and observation. Digital images of type specimens of the genus *Oreocharis*, available at the JSTOR Global Plants (http://plants.jstor.org/), were thoroughly examined. Additionally, collections (actual specimens or digital photos) housed in the herbaria BM, E, NE, GH, KUN, and YUKU (following acronyms standardized by Thiers (2025) [updated continuously]) were carefully reviewed and compared to the new species. The pertinent taxonomic literature (Wang et al. 1990, 1998; Wei et al. 2010; Möller 2019; Wen et al. 2025; GRC 2025) was consulted extensively to ensure that no known species were overlooked.

## ﻿Taxonomic treatment

### ﻿ Oreocharis corallodiscoides

Taxon classificationPlantaePasseriformesParamythiidae

Huan C. Wang & Xi Li sp. nov.

FFB736EB-761B-5A03-AD01-E88F2AA8A251

urn:lsid:ipni.org:names:77361684-1

Figs 1 , 2 , 3

#### Diagnosis.

*Oreochariscorallodiscoides* is similar to *O.agnesiae* (Forrest ex W. W. Sm.) Mich. Möller et W. H. Chen in texture and indumentum of the leaves, as well as in flower morphology and size, but can be clearly distinguished from the latter by its leaves ovate, rounded or sometimes rhombic (vs. ovate to oblong), with a length-to-width ratio of 1–1.5 (vs. 2.2–2.7), corollas yellow (vs. purple-red), and the presence (vs. absence) of two purple and triangular appendages inside the base of the corolla tube. Additionally, while *O.corallodiscoides* shares the inflated yellow corolla tube with *O.longifolia*, it differs by its ovate-rotund leaves (vs. lanceolate-oblong), shorter petioles (0.5–2 cm vs. 3.5–5 cm), crenate leaf margins (vs. serrate), glandular pistils (vs. glabrous), and the presence of appendages with thickened filaments.

#### Type.

China • Yunnan province, Yimen county, Luzhi town, near Xiaoluzhi village, 24°40'N, 101°57'E, 1300–1400 m a.s.l., 25^th^ Sept. 2015, *H. C. Wang et al. YM239* (Holotype: YUKU [2074890]!; isotypes: KUN, YUKU).

#### Description.

***Herbs perennial*.** Rhizome inconspicuous, with numerous fibrous roots. ***Leaves*** 9–18 in basal rosette, petiolate; ***petiole*** complanate, 0.5–2 cm long, both surfaces with densely appressed multicellular hairs; ***leaf blade*** ovate, rotund, or sometimes rhombic, 2.2–3.5 × 1.1–2.2 cm, coriaceous when dried; apex rounded, base broadly cuneate to rounded, margins crenate, adaxial surfaces densely white to gray strigose, abaxial with densely appressed multicellular hairs; ***lateral veins*** 4 or 5 on each side of midrib, adaxially inconspicuous, abaxially conspicuous. ***Cymes*** 1–2 (–4), scapiform, 1-flowered; ***peduncles*** 4–7 cm long, with dense glandular hairs; ***bracts*** 2, inserted above middle of cymes, elliptic to lanceolate, 2.0–4.0 × ca. 1.0 mm, with entire margins, outside with glandular hair, inside nearly glabrous; ***pedicels*** 2–3 cm long, with dense glandular hairs. ***Calyx*** 5-parted to near base, narrowly triangular, apex acute, margin entire, outside with glandular hair, inside nearly glabrous, sepals unequal, 4–6 mm long. ***Corolla*** yellow, not spotted or striped, 2.0–3.5 cm long, ca. 1–1.2 cm wide at mouth, outside covered with glandular hairs, glabrous inside; ***tube*** 1.5–2.5 cm long, inflated, infundibuliform and slightly curved downwards, two triangular purple appendages attached to base of tube, ca. 3 mm long, 2 mm wide at the base, apex acute; ***limb*** 5–6-lobed, distinctly 2-lipped, adaxial lip 4–6 mm long, unlobed, apex with 3 teeth, abaxial lip 7–8 mm long, 3-lobed, lobes wide triangular, 3–4 mm wide, apex acuminate; ***disk*** ring-like, ca. 1 mm in height; ***fertile stamens*** 4, adnate to corolla base, coherent in pairs, included, filaments ca. 2 cm in length; ***staminode*** 1, ca. 1 cm in length, with glandular hairs, degenerate anthers reniform. ***Pistil*** with glandular hairs, 2–2.5 cm long, ovary long cylindrical, light yellow, 1-loculed, style 1–2 mm long, stigma bilobed. ***Capsule*** oblanceolate-oblong, 4–6 cm long, commonly one side of the capsule dehiscing first.

**Figure 1.**
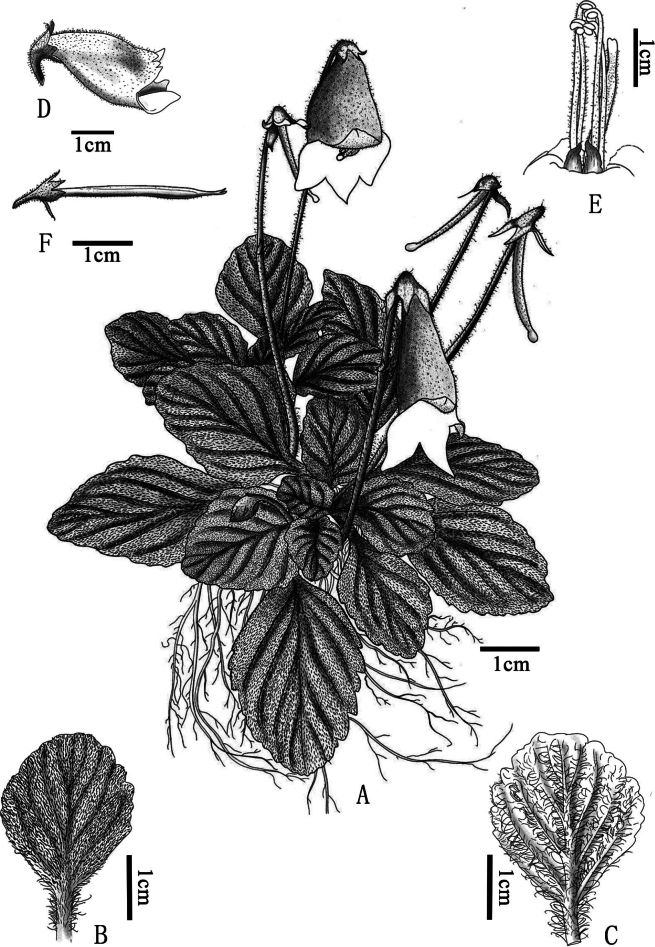
*Oreochariscorallodiscoides* sp. nov. **A** habit **B** adaxial surface of leaf **C** abaxial surface of leaf **D** flower (side view) **E** style, stamens and two triangular purple appendages **F** immaturate capsule.

#### Phenology.

*Oreochariscorallodiscoides* was observed flowering from August to September, and fruiting in October.

#### Etymology.

The specific epithet *corallodiscoides* is derived from the generic name *Corallodiscus* Batalin (Gesneriaceae) by adding the suffix “-oides”, reflecting the leaf morphological similarity of this new species to some members of the genus *Corallodiscus*, such as *C. lanuginosus* (Wall. ex A. DC.) B. L. Burtt; particularly, its sometimes rhombic leaves are rare in the genus *Oreocharis*.

**Figure 2.**
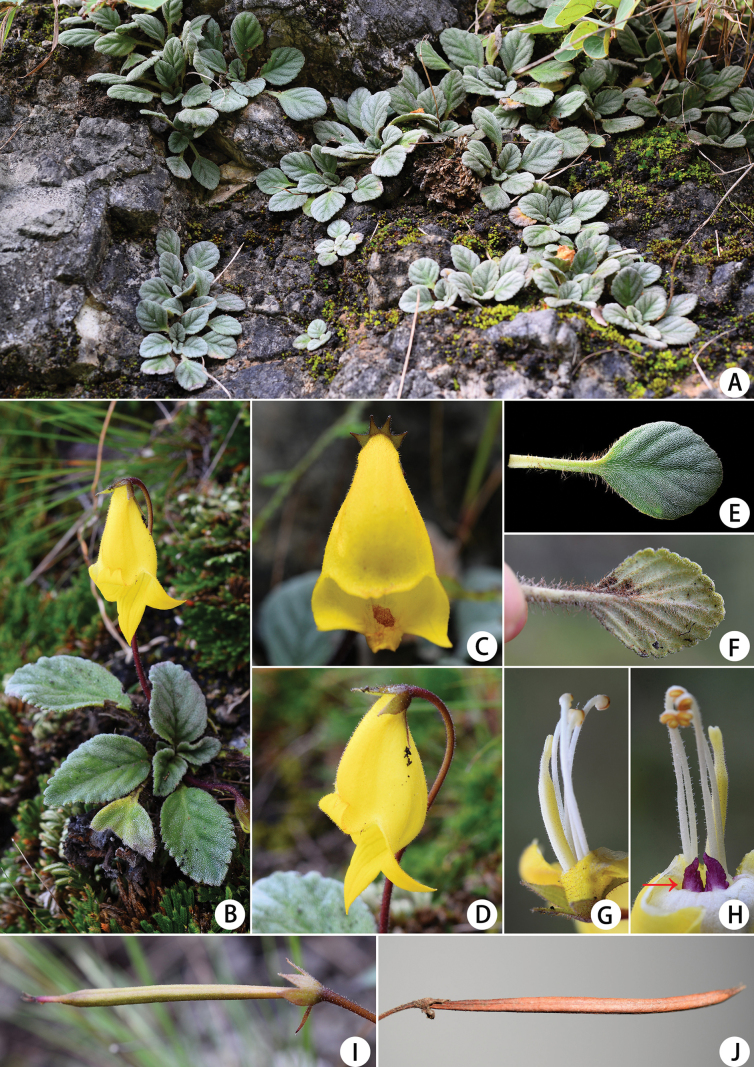
*Oreochariscorallodiscoides* sp. nov. **A, B** habit **C** flower in front view **D** flower in side view, pedicel **E** adaxial surface of leaf **F** abaxial surface of leaf **G** pistil and stamens **H** pistil, stamens and two triangular purple glandular appendages (at the arrow) **I** immaturate capsule **J** mature capsule.

#### Vernacular name.

Shan hu ye fo du ju tai (珊瑚叶佛肚苣苔) (Chinese).

#### Distribution and ecology.

**Figure 3.**
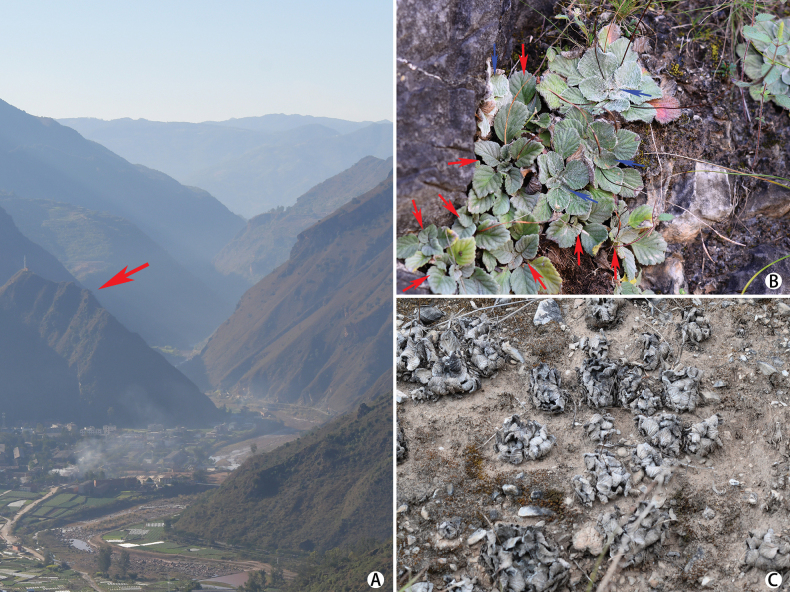
Habitat of *Oreochariscorallodiscoides* sp. nov. **A** overview of the Luzhijiang valley showing the type locality (indicated by red arrow) **B** close-up of the fruiting habitat (*Oreochariscorallodiscoides* and *Corallodiscuslanuginosus* are indicated by red and blue arrows respectively **C** close-up of the xeric habitat during the dry season.

*Oreochariscorallodiscoides* is known only from Xiaoluzhi village in the Luzhijiang valley, Yimen county, Yunnan province, southwest China. Its habitat is characterized by seasonally hot and arid conditions, occurring on dry slopes within the valley (Fig. 3). The species is typically found in limestone grasslands at elevations ranging from 1,300 to 1,400 meters, where water availability is limited. Additionally, *Oreochariscorallodiscoides* often grows together with *Corallodiscuslanuginosus* (Wall. ex A. DC.) B. L. Burtt, and interestingly, these two species of different genera have very similar leaf shapes and indumentum (Fig. 3B).

#### Conservation status.

*Oreochariscorallodiscoides* is a rare species with a restricted geographical range and a small population size. It is currently known only from the upstream region of the Luzhijiang river in Yimen county, an area that lies outside any officially protected zone. Based on the IUCN Red List Categories and Criteria (Version 16) (IUCN, 2024), the species meets Criterion D (very small or restricted population), with total mature individuals fewer than 250 mature individuals. Thus, this newly described species is preliminarily assessed as Endangered (EN).

## ﻿Discussion

Given its habitat and morphological characteristics, *Oreochariscorallodiscoides* appears to be a xerophytic plant well adapted to arid environments. This new species has leathery, thick-textured leaves densely covered with an indumentum and characterized by a relatively small leaf area. Unlike many congeners that grow on damp, shady rocks or cliffs in forested or rocky areas (Wei et al. 2010), *O.corallodiscoides* is found on open, dry, rocky slopes within dry-hot valleys. Additionally, the presence of two purple, triangular appendages at the base of the corolla tube (Fig. 2H) represents a character unique to this species, as it has not been observed in other members of the genus by us or documented in existing literature.

**Figure 4.**
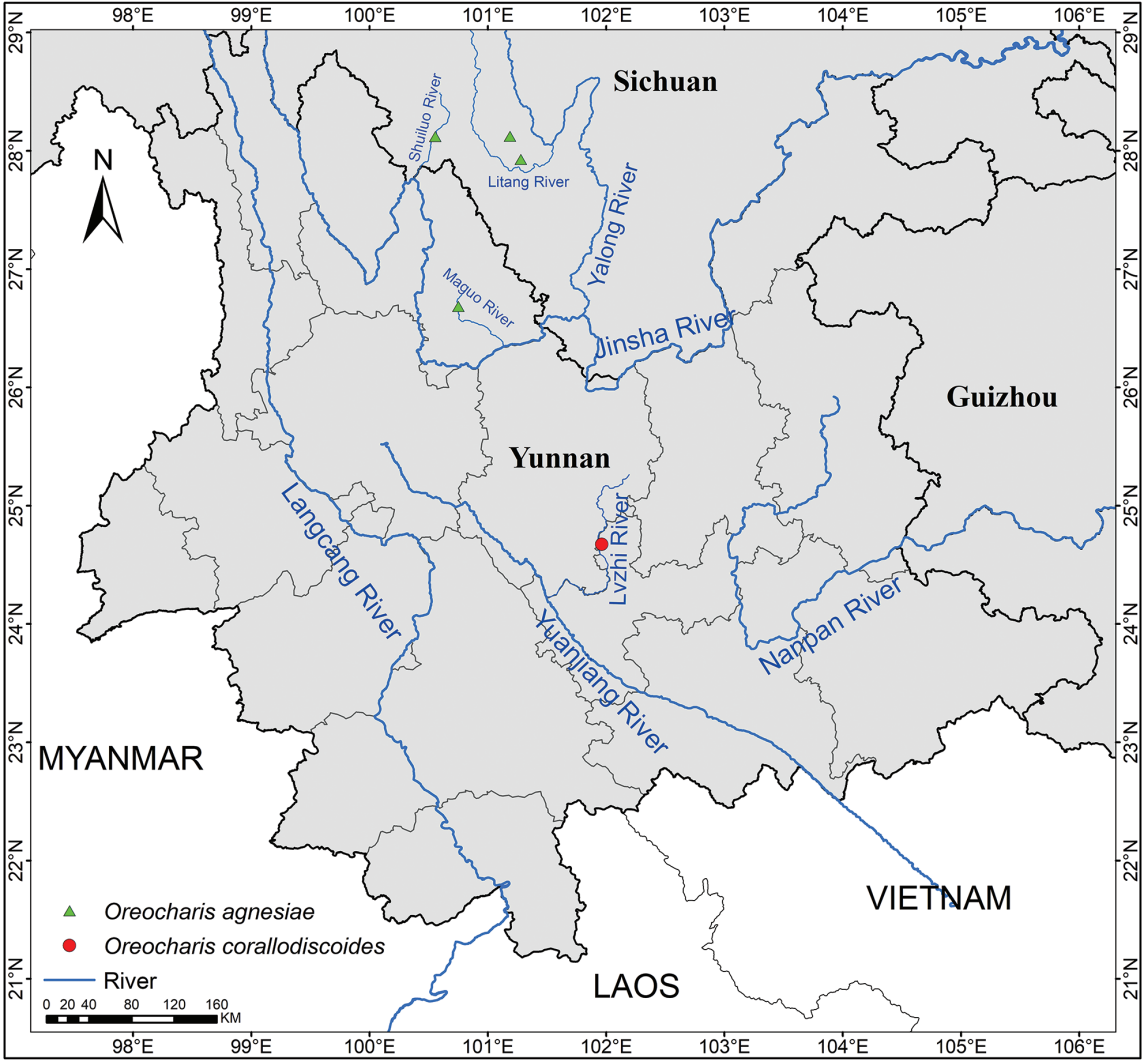
Geographic distribution of *Oreochariscorallodiscoides* and its morphologically similar species *O.agnesiae*.

Morphologically, *Oreochariscorallodiscoides* resembles *O.agnesiae*, sharing similar leaf indumentum and flower morphology. However, these two species show significant differences in corolla color: *O.corallodiscoides* has a uniformly yellow corolla, whereas the corolla of *O.agnesiae* is purple. Additionally, their leaf morphology also differs markedly. Geographically, *O.corallodiscoides* has a distinct and non-overlapping distribution range with *O.agnesiae* (Fig. 4). *Oreochariscorallodiscoides* is restricted to the Luzhijiang valley, an upstream tributary of the Yuanjiang river, at elevations of 1,300–1,400 m in central Yunnan. In contrast, *O.agnesiae* is distributed in the valleys of the Jinsha river and its tributaries at elevations of 1,900–3,000 m in northwestern Yunnan and southwestern Sichuan (Fig. 4). *Oreochariscorallodiscoides* is morphologically similar to *O.longifolia* (Craib) Mich. Möller et A. Weber in sharing inflated corolla tube and yellow color. However, *O.corallodiscoides* obviously differs from *O.longifolia* by its leaf blade shape (ovate to rotund vs. lanceolate to oblong), petiole length (0.5–2 vs. 3.5–5 cm), leaf length (2.2–3.5 vs. 8–23 cm), leaf margin (crenate vs. serrate), indumentum of pistil (glandular hair vs. glabrous), appendages (presence vs. absence) and filaments (thickened vs. not thickened).

**Additional specimens examined (Paratypes). China** • **Yunnan**: Yimen County, Luzhi town, Xiaoluzhi village, elev. ca. 1340 m, 22 Oct. 2015, H. C. Wang et al. YM324 (YUKU).

## Supplementary Material

XML Treatment for Oreocharis corallodiscoides

## ﻿Additional information

### Author ORCIDs

Xi Li https://orcid.org/0009-0003-8498-7809

Feng Yang https://orcid.org/0000-0003-2076-0130

Jing-Yi Ye https://orcid.org/0000-0002-3737-0115

Huan-Chong Wang https://orcid.org/0000-0001-8562-8849

### Data availability

All of the data that support the findings of this study are available in the main text.
